# Supplementary material for: Point-Prevalence Survey of Antimicrobial Use in Benin Hospitals: The Need for Antimicrobial Stewardship Programs
Source: Antibiotics (Basel). 2025 Jun 18;14(6):618. doi: 10.3390/antibiotics14060618 (PMC12189640; doi:10.3390/antibiotics14060618)
Supplement: Supplementary file 1 [file antibiotics-14-00618-s001.zip › antibiotics-3654399-SI.pdf]

Supplementary Materials

Section S1: Paper forms for the patient PPT survey and for the care units or sub-units adapted from the Global-PPS [17]

GLOBAL-PPS PATIENT

CU and hospital name	Activity (M, S, IC)	Name/First Name patient (date of birth)	Identification No<br>BEN – « hospital » - Care Unit – chronological number (R)	Patient age: Years ≥ 2 years	Patient age: Months 1-23 months	Patient age: Days <1 month	*Current weight* (kg)*	*Neonatal: Gestation age*	*Neonatal: Birth weight (kg)*	Sex M, F, U

**Symptoms presented or reason for consultation on survey day** (multiple choice, max. 6 choices)	**O** Fever >=38.3°C **O** Low-grade fever temperature (37.5-38.2°C) **O** Sneezing/nasal congestion **O** Runny or blocked nose **O** Cough **O** Sore throat **O** Dyspnea, breathing difficulties **O** Musculoskeletal pain **O** Headache **O** Fatigue/lethargy **O** Seizures **O** Earache **O** Diarrhea **O** Bloody diarrhea **O** Painful/frequent urination **O** Abdominal pain **O** Nausea/vomiting **O** Skin lesion/spot/wound **O** Itching or other genital/anal symptoms **O** Other symptom(s) **O** Unknown **O** None, other reason (e.g. prophylaxis)<br>**Note :**

**Admission status**	**O** Already admitted **O** Presumed admission **O** Referral to another institution (_ _ _ _ _ _ _ _ _ _ _ _ _ _ _ _ _) **O** Home **O** UNK

To be completed only if the patient receives a systemic antimicrobial during the defined time slot on the survey day.

**Treatment based on biomarker data :**				0 Yes – 0 No		**Culture(s) sent to the lab to document infection (Select if yes)**		
**If yes, which biomarker:** CRP, PCT, white blood cells count		**Biological sample type** (Blood/Urine/ Other)		**Most relevant value near the start of antibiotic therapy**<br>Value	Unit	☐ **Blood**	☐ **Cerebrospinal fluid**	☐ **Bronchoalveolar lavage**
						☐ **Urine**	☐ **Wound (surgery/biopsy)**	☐ **Sputum/Bronchial aspirations**
						☐ **Stool**	☐ **Other sample type**	

**Renal function analysis**	0 Yes – 0 No	**Type of calculation**	_ _ _ _ _ _ _ _ _ _ _ _ _ _ _ _ _ _ _	**Value (+ Unit)**	

**Treatment based on rapid diagnostic test**	**0** Yes **0** No
**If yes, specify**	
**Results, specify**	**0** Positive **0** Negative **0** Inconclusive

**Underlying morbidity** *(max. 3 choices)*	**O** Diabetes mellitus, type 1 or 2	**O** Trauma	**O** Chronic / Long COVID	**O** HIV
	**O** Hematologic or solid cancer/ Recent chemotherapy (< 3 months)	**O** Non oncological, immunocompromised	**O** Gastrointestinal disease: inflammatory bowel disease, celiac disease	**O** Chronic pulmonary diseases, cystic fibrosis, COPD, bronchiectasis, asthma
	**O** Malnutrition	**O** Other	**O** None	**O** Unknow

**Antimicrobial** (generic) **Name**	**1.**	**2.**	**3.**	**4.**	**5.**
**AWaRe category according to WHO** (Acces, Watch, Reserve)					
**Prescriber name/type of prescriber if known**					
**Specify by prescription : new** or **ongoing**					
**Unit dose** / **Unit** (g, mg, UI, UM)					
**Doses/day** / **Route of administration** (O, R, I, IM, IV)					
**Adjustment for renal function ? (not 1st dose)**	0 Yes 0 No (high)<br>0 NE 0 No (low)	0 Yes 0 No (high)<br>0 NE 0 No (low)	0 Yes 0 No (high)<br>0 NE 0 No (low)	0 Yes 0 No (high)<br>0 NE 0 No (low)	0 Yes 0 No (high)<br>0 NE 0 No (low)
**Prescribed/Planned therapy duration in N days/ UNK**					
**End date/ Reassessment documented in the file ?**	0 Yes 0 No	0 Yes 0 No	0 Yes 0 No	0 Yes 0 No	0 Yes 0 No
**Clinical diagnosis** (Table 2 protocol)					
**Type of indication** (Table 3 protocol)					
**Diagnosis/Indication documented in the file?**	0 Yes 0 No 0 UNK	0 Yes 0 No 0 UNK	0 Yes 0 No 0 UNK	0 Yes 0 No 0 UNK	0 Yes 0 No 0 UNK
**Infectiologist opinion?**	0 Yes 0 No 0 UNK	0 Yes 0 No 0 UNK	0 Yes 0 No 0 UNK	0 Yes 0 No 0 UNK	0 Yes 0 No 0 UNK
**Local directive existence for diagnosis ?**					
**If yes (existing directive) :**<br>**Drug compliant with directive ?** (Y, N, NE, U)					
**Reason ?** (Antibiotic spectrum too broad or too narrow? Antimicrobial unnecessary ?)					
**Dosage compliant with directive ?** (Y, N, NE, U)					
**Duration compliant with directive ?** (Y, N, NE, U)					
**Comments**					

**Treatment (E: Empirical; T: Targeted)**										
**The following resistance data is to be filled in only if the treatment choice is based on microbiology data (Treatment=T) available on the day of the PPS**										
**Maximum 3 microorganisms (MO) to report**<br>**Maximum 1 resistance type per MO to report**	**MO**	**R type****	**MO**	**R type****	**MO**	**R type****	**MO**	**R type****	**MO**	**R type****
**Indicate codes** **MO 1**										
**MO 2**										
**MO 3**										

Resistance type**- choose between: **MRSA**[19]; **MRCoNS**[20]; **PNSP**[21]; **MLS**[22]; **VRE**[23]; **ESBL** (ESBL-producing Enterobacterales[24]); **3GCREB** (3rd generation cephalosporin resistant Enterobacterales); **CRE** (Carbapenem-resistant Enterobacterales[25]); **ESBL-NF** (ESBL-producing non fermenter Gram-negative bacilli[26]); **CR-NF** (Carbapenem-resistant non fermenter Gram-negative bacilli [27]); **other MDRO**[28]; **Azoles**[29].

[19] Methicillin-resistant *Staphylococcus aureus* (MRSA), [20] Methicillin-resistant coagulase negative staphylococci (MRCoNS), [21] Penicillin-non susceptible *Streptococcus pneumoniae* (PNSP), [22] Macrolide-lincosamide-streptogramin resistance in Streptococcus isolates (MLS), [23] Vancomycin-resistant enterococci (VRE), [24] Bacteria, producing extended-spectrum beta-lactamases (ESBL), [25] Carbapenem-resistant *Enterobacterales* (CRE) – enteric bacteria resistant to imipenem, meropenem or other carbapenems, [26] ESBL Non fermenters (ESBL-NF): *Pseudomonas aeruginosa, Acinetobacter baumannii, Burkholderia spp., Stenotrophomonas maltophilia* multidrug resistant, [27] Carbapenem-resistant Nonfermenters (CR-NF) – nonfermenters resistant to imipenem, meropenem or other carbapenems, [28] Multi-drug resistant (MDR) pathogens, others than the listed above, [29] Azoles to treat infections caused by azole-resistant fungi and yeasts (e.g. *Candida spp., Aspergillus spp*.

Adapted Global-PPS Audit: BENIN February/March 2024

**Study dates** (dd/mm/year)	_____/_____/______ to _______/______/	**Person completing form** (Auditor code) :	
**Hospital Name :**		**Name of the Care Unit (CU) :**	

	Adult CU		Pediatric CU
**Type of Care Unit :**<br>Select the most appropriate department for the CU	▫ **AMW** (General or mixed **Adult Medical Ward**)<br>▫ **HO-AMW** (Haematology-Oncology)<br>▫ **T-AMW** (Transplant (BMT/solid))<br>▫ **P-AMW** (Pneumology)<br>▫ **CAR-AMW** (Cardiology)<br>▫ **NEU-AMW**(Neurology)<br>▫ **REN-AMW** (Nephrology)<br>▫ **ID-AMW** (Infectious Disease)<br>▫ **DB-AMW** (Dermatology-burn wards)<br>▫ **PSY-AMW** (Psychiatry)<br>▫ **REH-AMW** (Rehabilitation)<br>▫ **GER-AMW** (Geriatrics)<br>▫ **LTC-AMW** (Long-Term care)<br>▫ **OBG-AMW** (gynaecology-obstetrics)<br>▫ **IS-AMW** (Isolation ward, COVID patients)	▫ **ASW** (General or mixed **Adult Surgical Ward**)<br>▫ **DIG-ASW** (Digestive tract surgery)<br>▫ **ORT-ASW** (Orthopaedics-Trauma surg.)<br>▫ **URO-ASW** (Urological surg.)<br>▫ **CV-ASW** (Cardio-vascular surg.)<br>▫ **NEU-ASW** (Neurosurgery)<br>▫ **ONCO-ASW** (Oncology-cancer surg.)<br>▫ **PLAS-ASW** (Plastic, reconstructive surg.)<br>▫ **ENT-ASW** (Ear-nose-throat surg.)<br><br>▫ **AICU** (General or mixed **Adult Intensive Care Unit)**<br>▫ **MED-AICU** (Medical AICU)<br>▫ **SUR-AICU** (Surgical AICU)<br>▫ **CAR-AICU** (Cardiac AICU)<br>▫ **AHDU** (High Dependency Unit)	▫ **PMW** (Paediatric Medical Ward)<br>▫ **HO-PMW** (Haematology-Oncology)<br>▫ **T-PMW** (Transplant (BMT/Solid))<br>▫ **PSW** (Paediatric Surgical Ward)<br>▫ **PICU** (Paediatric Intensive Care Unit)<br>▫ **ID-PMW** (Infectious Disease PMW)<br><br>**Neonatal wards:**<br>▫ **NMW** (Neonatal Medical Ward)<br>▫ **NICU** (Neonatal Intensive Care Unit)
**Mixed care unit**	▫ **Yes** ▫ **No**		

**Activity :**	▫ **Medicine**	▫ **Surgery**	▫ **Intensive Care**
**Total number of beds** available in the Care Unit. For mixed CUs, indicate the total number of beds corresponding to each section.			

Section S2: List of average prices of various AM prescribed in Benin between February and March 2024, according to SOBABS, the ABRP price catalogue and the main wholesalers of the Hévié pharmacy (21, 22, 23).

Table S1: Average prices of various systemic antibacterials (J01) prescribed in Benin

Therapeutic class	International Non-proprietary Name (INN)	Oral route	Price U (FCFA)	Intravenous route	Price U (FCFA)
Aminoglycosides	Gentamicin	-	**-**	80 mg/2 mL	**200**
Aminopenicillin/beta-lactamase inhibitor	Amoxicillin/Ac. Clavulanic	500/62,5 ; 875/125 ; 1000/125 mg	**203**	500/100 ; 1000/200 mg	**1635 - 2720**
	Piperacillin/Tazobactam	-	**-**	4000/500 mg	**4038**
	Cefadroxil	500 mg	**465**	-	**-**
2nd generation cephalosporin	Cefuroxime	500 mg	**302**	750 mg	**1665**
3rd generation cephalosporin	Cefixime	200 mg ; 400 mg	**413**	-	**-**
	Cefotaxime	-	**-**	1000 mg	**2670**
	Cefpodoxime	100 mg ; 200 mg	**256**	-	**-**
	Ceftazidime	-	**-**	1000 mg	**8173**
	Ceftriaxone	-	**-**	1000 mg	**1750**
3rd generation cephalosporin + penicillinase inhibitor	Ceftriaxone/Sulbactam	-	**-**	1000/500 mg	**2920**
4th generation cephalosporin	Cefepime	-	**-**	1000 mg	**2622**
Carbapenem	Meropeneme	-	**-**	500 ; 1000 mg	**5841**
Carbapenem + dihydropeptidase-1 inhibitor	Imipeneme/Cilastatine	-	**-**	500/500 mg	**4012**
Lincosamide	Lincomycin	500 mg	**108**	600 mg/2 mL	**1675**
Macrolides	Azithromycin	250 ; 500 mg	**511**	-	**-**
	Clarithromycin	500 mg	**223**	-	**-**
	Clindamycin	150 ; 300 mg	**70**	-	**-**
	Erythromycin	500 mg	**40**	-	**-**
	Josamycin	500 mg	**295**	-	**-**
	Roxithromycin	150 mg	**295**	-	**-**
	Spiramycin	1,5 ; 3 MUI	**459**	-	**-**
Macrolide + Nitro-5-Imidazole	Spiramycin/Métronidazole	1,5 MUI/250 mg	**177**	-	**-**
Nitro-5-imidazole	Metronidazole	250 ; 500 mg	**41**	500 mg/100 mL	**2670**
	Secnidazole	1000 mg	**256**	-	**-**
Nitroimidazole + Antifungal + Macrolide	Secnidazole/Fluconazole/Azithromycin	1000/150/1000 mg	**465**	-	**-**
Penicillin	Amoxicillin	500 ; 1000 mg	**98**	-	**-**
	Ampicillin	500 mg	**30**	500 ; 1000 mg	**100 - 300**

	Cloxacillin	250 ; 500 mg	**46**	500 ; 1000 mg	**165 - 225**
	Flucloxacillin	500 mg	**52**	500 ; 1000 mg	**1375 - 2335**
	Benzyl Pénicillin	-	**-**	0,6 ; 1,2 et 2,4 MUI	**1155 - 1470**
Quinolone	Ciprofloxacin	250 ; 500 ; 750 mg	**367**	200 mg/100 mL	**1050**
	Levofloxacin	250 ; 500 ; 750 mg	**302**	500 mg/100 mL	**3999**
	Moxifloxacin	400 mg	**1635**	-	**-**
	Norfloxacin	400 mg	**443**	-	**-**
	Ofloxacin	200 ; 400 mg	**315**	200 mg/40 mL	**1400**
Quinolone + Nitro-5-imidazole	Ciprofloxacin/Tinidazole	500/600 mg	**602**	-	**-**
	Ofloxacin/Ornidazole	200/500 mg	**229**	-	**-**
	Norfloxacin/Métronidazole	200/200 mg	**174**	-	**-**
Sulfamides	Sulfamethoxazole/Trimethoprime	400/80 mg ; 800/160 mg	**210**	-	**-**
Tetracycline	Tetracycline	250 mg	**270**	-	**-**
	Doxycycline	100 ; 200 mg	**202**	-	**-**
Other antibacterials	Fosfomycin	3 g	**5955**	-	**-**

Ac. Clavulanic: Clavulanic acid; FCFA: Francs de Colonies Françaises Africaines (currency in Benin); U: Unit.

N.B.: Oral forms in drinkable suspension are not included in this price analysis table. These are public prices.

The daily cost of antimicrobial therapy is highly dependent on the prescribed dosages, which vary based on the severity of infection and whether recommended dosing guidelines are strictly followed or not. Therefore, including a uniform daily price may not reflect the actual clinical practice and could be misleading. The actual daily cost should be calculated based on each prescription's dose regimen (hence the inclusion of the unit price per formulation in the table s1).

Section S3: Infectious conditions diagnosed by clinicians.

Table S2: Main infectious diagnoses at hospital A (N = 92) and B (N = 120)

	**Hospital A**	**Hospital B**	**Total**
	N (%)	N (%)	N (%)
Bacteremia	0 (0)	0 (0)	0 (0)
Heart infections	0 (0)	0 (0)	0 (0)
Gastrointestinal infections	41 (45)	11 (9)	52 (25)
Gynecological and obstetric infections	3 (3)	27 (23)	30 (14)
Neonatal infections	0 (0)	0 (0)	0 (0)
ENT Infections	2 (2)	0 (0)	2 (1)
Skin and Soft-tissue Infections	28 (30)	1 (1)	29 (14)
Respiratory Infections	6 (7)	1 (1)	7 (3)
CNS Infections	0 (0)	0 (0)	0 (0)
Urinary tract infections	2 (2)	7 (6)	9 (4)
Eye Infection	0 (0)	0 (0)	0 (0)
Malaria	6 (7)	6 (5)	12 (6)
Gynecological-obstetric prophylaxis	0 (0)	64 (53)	64 (30)
Sepsis	6 (7)	4 (3)	10 (5)
HIV	0 (0)	0 (0)	0 (0)
Other	0 (0)	0 (0)	0 (0)
Unknown	4 (4)	3 (3)	7 (3)

ENT: Ear, Nose and Throat; CNS: Central Nervous System; HIV: Human Immunodeficiency Virus.

The various prescriptions for sepsis (10) were analyzed and categorized into the corresponding infections: 4 gynecological-obstetric infections, 3 pulmonary infections, 2 skin and soft-tissue infections, and 1 gastrointestinal infection. The 7 prescriptions with unknown diagnoses included one case each of intrauterine fetal demise, abdominal pain, vaginal discharge, stroke sequelae associated with epileptic seizures, asthenia, and two cases of diabetic ketoacidosis.

Section S4: Antimicrobial treatments prescribed for postoperative gynecological-obstetric prophylaxis lasting more than 1 day (SP3) at hospital B (N = 26).

Table S3: AM prescribed for post-operative gynecological-obstetric prophylaxis SP3 (N = 26)

	**Hospital B**
	N (%)
Amoxicillin/Clavulanic Acid	1 (4)
Cefuroxime	1 (4)
Metronidazole	10 (38)
Metronidazole + Cefuroxime	8 (31)
Metronidazole + Amoxicillin	1 (4)
Metronidazole + Amoxicillin/Clavulanic Acid	5 (19)

AM : Antimicrobials; SP3 : Surgical prophylaxis > 1 day.

Section S5: Analysis of quality and compliance indicators for MA prescriptions in the two hospitals according to the diagnoses of the two main infections.

Table S4: Gastrointestinal infections (GI) and skin and soft-tissue infections at hospital A - Quality and Compliance Indicators for MA Prescriptions

	Infections GI<br><br>N (%)	Skin/soft-tissue infections<br><br>N (%)
Prescriptions with documented indication	41	28
Choice of compliant AM (if documented indication)	19 (46)	11 (39)
Spectrum too large	6 (27)	3 (18)
Spectrum too narrow	2 (9)	14 (82)
MA not required	14 (64)	0 (0)
Dosage compliance	17 (41)	7 (25)
Prescriptions with a documented stop/review date	16 (39)	24 (86)
Duration compliance (if stop date/reassessment documented)	12 (75)	21 (88)

MA : Antimicrobials

The two main diagnoses involved gastrointestinal infections (41/92; 45%) and skin and soft-tissue infections (28/92; 30%). Among the 41 documented AM prescriptions for gastrointestinal infections, 25 (61%) were for digestive infections, 15 (37%) for intra-abdominal infections and sepsis, and 1 (2%) for digestive surgical prophylaxis. Regarding adherence to guidelines for AM choice, only 46% (19/41) of these prescriptions were compliant with international guidelines. Of the 22 non-compliant prescriptions, 14 (64%) were unnecessary (e.g., AM for food poisoning without alarm signs); 6 (27%) had too broad a spectrum (e.g., Amoxicillin/Clavulanic Acid with Metronidazole for gastritis); and 2 (9%) had too narrow a spectrum (e.g., Ofloxacin or Norfloxacin alone for liver abscesses). In terms of dosage compliance, only 17 out of 41 prescriptions (41%) adhered to international guidelines. Prescription durations were largely compliant, with 75% (12/16) meeting the standards.

For skin and soft-tissue infections, all 28 documented AM prescriptions were exclusively for skin, soft-tissue, and surgical-site infections. Only 39% (11/28) of these prescriptions were compliant with

guidelines for AM choice. Among the 17 non-compliant prescriptions, 14 (82%) had too narrow a spectrum (e.g., Lincomycin with Metronidazole for traumatic wounds); and 3 (18%) had too broad a spectrum (e.g., Ceftriaxone for traumatic wounds). Regarding dosage compliance, only 7 out of 28 prescriptions (25%) met international guidelines. However, most of the prescription durations were compliant, with 88% (21/24) adhering to the guidelines.

Table S5: Gynecological and obstetric infections and prophylaxis at hospital B - Quality and compliance indicators for AM prescriptions

	Proph. OBGY<br>N (%)	Inf. OBGY<br>N (%)
Prescriptions with documented indication	64	27
Choice of compliant AM (if documented indication)	2 (3)	21 (78)
Spectrum too large	0 (0)	3 (50)
Spectrum too narrow	25 (40)	3 (50)
MA not required	37 (60)	0 (0)
Dosage compliance	24 (38)	13 (48)
Prescriptions with a documented stop/review date	40 (62)	8 (30)
Duration compliance (if stop date/reassessment was documented)	3 (8)	8 (100)

AM : Antimicrobials ; Inf. OBGY : Gynecological and obstetric infections; Proph. : Gynecological and obstetric prophylaxis.

The two main diagnoses were gynecological-obstetric prophylaxis (64/120; 53%) and gynecological-obstetric infections (27/120; 23%). Among the 64 documented AM prescriptions for gynecological-obstetric prophylaxis, 24 (38%) were preoperative, and 40 (62%) were postoperative. Only 3% (2/64) of these prescriptions adhered to international guidelines for AM choice. Notably, 37 non-adherent prescriptions (60%) were unnecessary (37/40 postoperative prescriptions), and 25 others (40%) had too narrow a spectrum (e.g., Ampicillin for all 24 preoperative prescriptions). In terms of dosage compliance, only 24 out of 64 prescriptions (38%) met international guidelines. Prescription durations were poorly compliant, with only 3 out of 40 (8%) being appropriate, as the remaining 37 were unnecessary postoperative prescriptions.

For gynecological-obstetric infections, 21 out of 27 documented AM prescriptions (78%) adhered to the guidelines for AM choice. Among the non-adherent cases, 50% (3/6) had too narrow a spectrum, and the other 50% (3/6) had too broad a spectrum. Regarding dosage compliance, only 13 out of 27 prescriptions (48%) were in line with international guidelines. However, prescription durations were perfectly compliant, with all 8 evaluated prescriptions (100%) adhering to the guidelines.
